# Multifunctional 2D materials for antiviral protection and detection

**DOI:** 10.1093/nsr/nwab095

**Published:** 2021-05-27

**Authors:** Daria V Andreeva, Kostya S Novoselov

**Affiliations:** Department of Materials Science and Engineering, National University of Singapore, Singapore; Department of Materials Science and Engineering, National University of Singapore, Singapore; National Graphene Institute, University of Manchester, UK; Chongqing 2D Materials Institute, China

Two-dimensional (2D) materials [1] with their high surface to volume ratio, flexibility, tunable electronic properties, and most importantly—the large variety of the properties covered by the members of the 2D family—have already found their way to many applications in very different areas of technology [2]. One of the most promising areas of applications for 2D crystals remains healthcare [3], where such materials have been considered for drug delivery, artificial tissue, biosensing and many other important technologies [4]. Among different biological usage of 2D materials—multifunctional antiviral coatings are probably the most interesting and timely because of the large demand for smart healthcare solutions caused by the COVID-19 pandemic [5,6].

Despite the variety of properties collectively covered by different members of the 2D material family, interaction with biological objects would require additional functionality. Thus, antiviral coatings need to be able to control the surface work function, be pH responsive, demonstrate switchable wetting among other properties to actively protect against viruses, and possibly even to help to eliminate those. Furthermore, such properties need to be adaptive to the external conditions. To this end, composite materials based on 2D crystals and polyelectrolytes might be very effective. 2D materials produce a solid, continuous and robust framework, with specific conductivity, optical adsorption and work function [7]. At the same time polyelectrolytes are known to change their characteristics depending on the immediate environment, adsorbing or desorbing various chemical species and water [8]. Thus, polyelectrolytes can change their charging state and their conformation in response to the changes in the chemical, physical and biological environments [8]. The combination of the adaptive nature of polyelectrolytes with the optical, electronic and mechanical properties of 2D materials might be extremely beneficial for antiviral applications. Furthermore, such a combination gives very interesting opportunities not only in protective applications but also for biological sensing.

**Figure 1.**
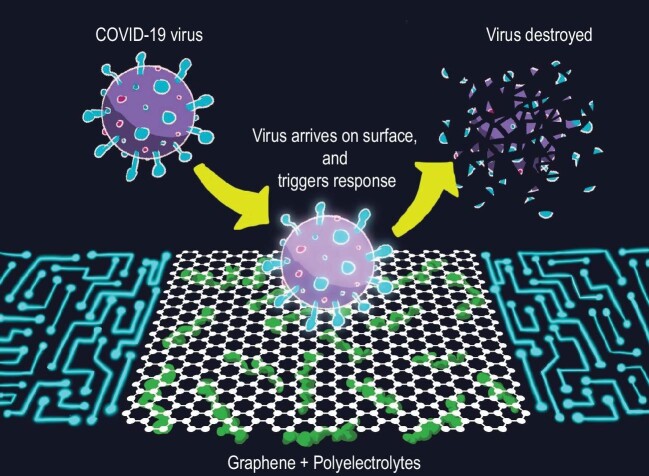
The new 3-in-1 transistors combine materials that can sense the presence of viruses, protect the surfaces against viruses and function as a substrate with switchable a circuit operating with ionic current. Such devices, together with information transmitters, energy harvesting and energy storage components, can be attached to flexible substrates and used for the assembly of smart healthcare devices.

The 2D composite membranes formed by self-assembly of negatively charged graphene oxide and positively charged polyelectrolytes (polycations) are capable of regulating pH and ionic gradients and, therefore, regulate ionic currents under a large range of external conditions. We believe that such structures will also be useful for antiviral applications. In particular, polycations (e.g. synthetic polyamines, chitosan, amino groups in proteins) have strong affinity to protons [9]—they can selectively bind protons and regulate their diffusion. The 2D composites of graphene oxide with polycations form positively charged surfaces (through protonation of polyelectrolyte molecules) and can interact with negatively charged viral proteins. On exposure to viruses, such composite materials can release protons from the interior of the structure into the environment and change the surface charge of the viruses. Thus, such 2D composite materials can affect the structure of viruses and actively fight viruses, providing a barrier as well as active antiviral protection (Fig. 1).

Furthermore, regulated charging of the polyelectrolytes as well as conductivity and gating properties of 2D materials can be used for sensing functionalities. For most biological sensors, a conversion from ionic to electronic currents is crucial—biological systems generally rely on ionic currents, whereas modern electronics deal with electrons. The efficient conversion, which allows fast and reliable detection of changes in the chemical, physical and biological environment, can be done via utilization of polyelectrolytes combined with 2D materials. Different polyelectrolytes can dope 2D materials to a different polarity. By interfacing such 2D materials doped with different polyelectrolytes, one can form ionic p–n junctions. A complex structure with several different layers which are differently charged, can be self-assembled into bipolar ionic transistors with intrinsic amplification of the signal and, therefore, high sensitivity of new devices. The change of ionic concentration in the interior of the composite material, because of the presence of the virus, can be used as gating for control of ionic currents in the transistor structures.

In particular, graphene oxide doped with different polycations can be used to form a variety of ionic conductors with ionic p–n junctions and then can be assembled into an array of ionic transistors. Figure 1 is a schematic representation of such transistors. The surfaces made of cationic polyelectrolytes will interact with biological objects, including COVID-19. Through electrostatic interactions between viruses and polyelectrolytes, we can achieve: (1) triggered release of protons from polyelectrolyte chains to compensate for the negative charge of virus surfaces; (2) a highly localized acidic environment, which can affect viral proteins and deactivate them (deactivation); and (3) switch of ionic current in the transistors (detection and signaling function). Such devices could be combined with information transmitters, energy harvesting and energy storage materials and can be attached to flexible substrates to create smart healthcare devices.

There are many other 2D materials beyond graphene which could be explored for use in formation of such self-assembled heterostructures for a variety of biological applications, including

antiviral and antimicrobial protection. MoS_2_, MoSe_2_, WS_2_, WSe_2_, MoTe_2_, InS, InSe, GaS, GaSe and hBN present a rich set of useful optical and electronic properties [7,10] for the construction of ionic devices controlled by gating. Semiconductors can generate photoinduced carriers upon illumination, changing their reduction or oxidation properties, opening up yet another avenue for remote control of antiviral and antimicrobial activities. Furthermore, as the band-gap of these materials (and thus the position of HOMO and LUMO orbitals) can be controlled by the number of layers—tunability towards a particular part of the spectrum is possible (transition metal dichalcogenides have a band gap energy ∼1.2–1.8 eV for instance) [7]. The selection of 2D heterostructures increases the range of possible devices, which might include temperature, light and pressure sensing.

In general, smart responsive composite nanomaterials, based on 1D and 2D materials, are capable of adaptation to biological environment (viruses, bacteria and fungi) in tandem with intrinsic sensing capabilities. To assemble such devices, broad expertise with 2D materials, colloid synthesis and characterization, colloid assembly, and stimuli-responsive polymers is required. Low-dimensional multifunctional coatings and surfaces that can detect a biological environment and prevent its undesirable invasion into our life do not aggressively suppress natural processes, rather they regulate the environment and adapt it to our needs. In future, smart composites could be created through the proposed platform and are applicable to a broad range of areas, including corrosion protection, medicine, filtration and wearable electronics for healthcare applications.

***Conflict of interest statement*.** None declared.
